# Esophageal Lichen Planus: Understanding a Potentially Severe Stricturing Disease

**DOI:** 10.1155/2017/5480562

**Published:** 2017-10-04

**Authors:** Bharat Rao, Abhishek Gulati, Blair Jobe, Shyam Thakkar

**Affiliations:** ^1^Department of Gastroenterology, Allegheny Health Network, Pittsburgh, PA, USA; ^2^Allegheny Center for Digestive Health, 1307 Federal Street, Suite 301, Pittsburgh, PA 15212, USA; ^3^Department of Surgery, Allegheny Health Network, Pittsburgh, PA, USA; ^4^Esophageal and Lung Institute, 4815 Liberty Avenue, Mellon Pavilion, Suite 158, Pittsburgh, PA 15224, USA

## Abstract

A 67-year-old woman with a long-standing history of recurrent dysphagia and esophageal strictures failed to respond to aggressive antireflux management. She required multiple dilations for symptomatic strictures that were discovered throughout the esophagus. Intralesional, topical, and systemic glucocorticoid therapies were utilized without resolution in symptoms. Several years after initial presentation, histopathology ultimately demonstrated lichenoid features and a diagnosis of esophageal lichen planus (ELP) was confirmed. However, as her symptoms had already become significantly disabling with severe strictures that carried an increased risk of endoscopic complications with dilation, she ultimately decided to undergo an esophagectomy for definitive treatment. Moreover, ELP may often go unrecognized for several years. Clinicians should consider ELP in the differential for dysphagia in middle- to elderly-aged women with or without a known history of lichen planus (LP) especially for those with findings of multiple or proximal strictures.

## 1. Introduction

Lichen planus (LP), an inflammatory disorder with an unknown etiology, commonly manifests in the oral mucosa and skin [1]. LP though may involve much less common sites including the esophagus [2]. It is not uncommon to take few to several years to correctly diagnose esophageal lichen planus (ELP) due to unfamiliarity with the disease, often nonspecific endoscopic and histopathology findings, and the possibility of ELP being the first manifestation of LP [3, 4]. We present our own experience of a patient with a severe case of ELP in order to further the understanding of this potentially chronic and debilitating stricturing disease. Important concepts to diagnosis ELP early in its course along with management strategies are reviewed.

## 2. Case Presentation

A 67-year-old woman presented with a long-standing history of dysphagia and recurrent strictures. She did not have typical heartburn or regurgitation symptoms consistent with reflux disease. Her medical history included psoriasis and hypothyroidism. Exam findings of oral lesions or skin findings consistent with LP were not identified.

Laboratory investigations were remarkable for a positive antinuclear antibody with titres of 1 : 320 in a homogenous pattern with other autoimmune markers being nonreactive. Hepatitis C antibody was also nonreactive. Histopathology specimens predominantly showed nonspecific findings of acute and chronic inflammation suggestive of reflux disease.

**Figure 1:**
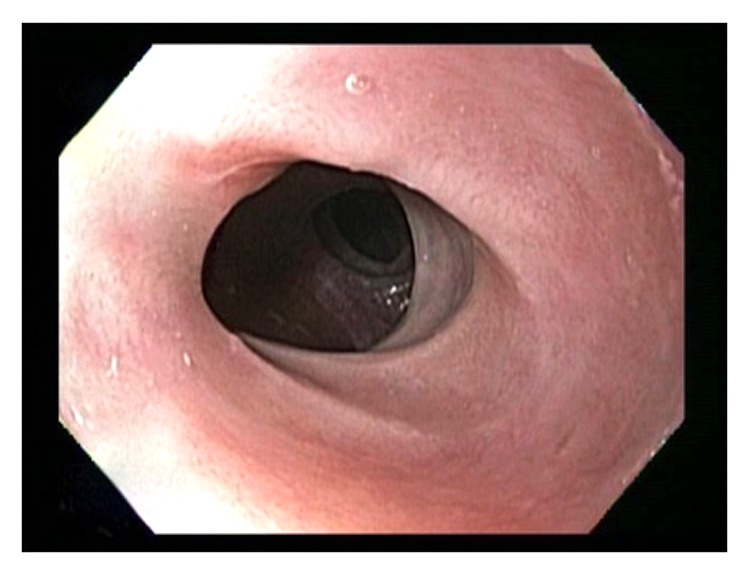
Stricture in the lower third of esophagus.

**Figure 2:**
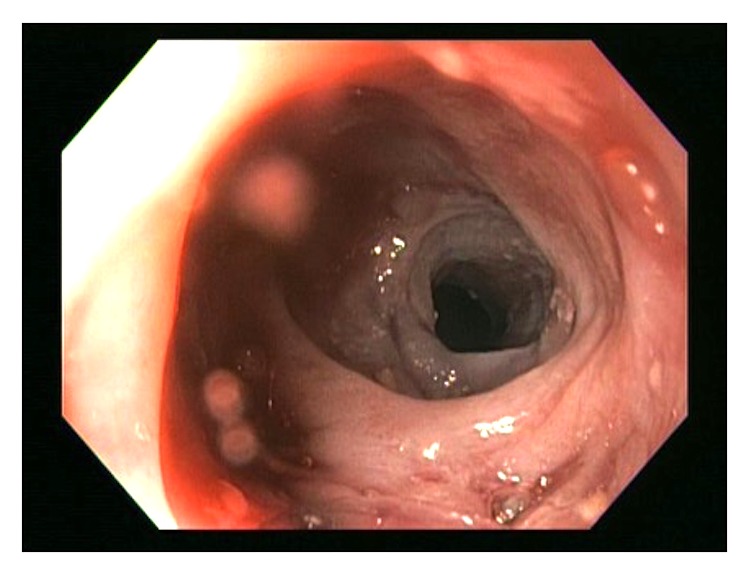
Stricture in the middle third of esophagus.

**Figure 3:**
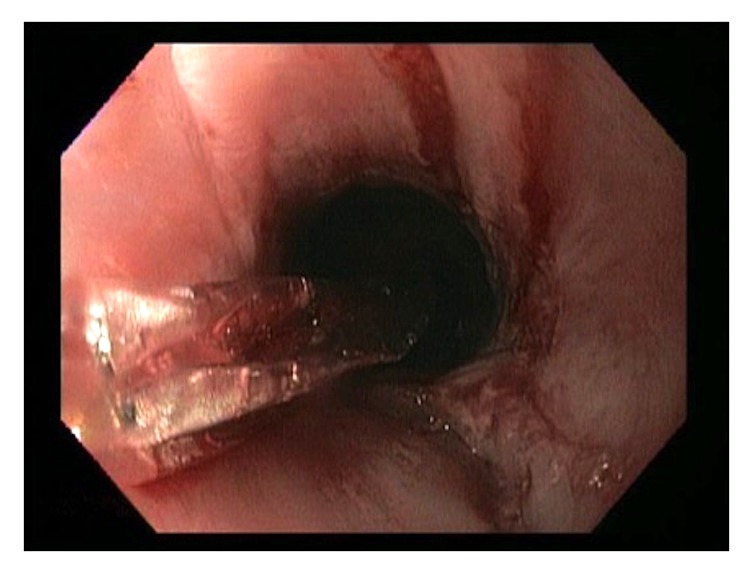
Proximal stricture treated with dilatation.

She underwent multiple esophagogastroduodenoscopies (EGD) with findings of mucosal friability, webs, and strictures (Figures 1–3). Initial examinations revealed strictures in the lower third of the esophagus. Later, strictures were discovered in both the proximal and mid-esophagus. Strictures ranged in diameter within 9–14 mm.

Initial treatment consisted of twice daily proton pump inhibitor (PPI) for initially suspected reflux esophagitis, which offered no improvement in symptoms. A trial of inhaled swallowed fluticasone propionate 440 mcg twice daily for at one point suspected eosinophilic esophagitis provided no relief. Endoscopic bougie and balloon dilations provided transient improvement but this became less sustained over time. The primary modality of treatment that offered prolonged benefit was recurrent dilations with triamcinolone acetonide injections (10 mg/mL concentration) to strictures though the time interval of relief ultimately decreased as well. A trial of systemic glucocorticoids (40 mg/daily with a tapered course over 10 weeks) was also given for a suspected autoimmune etiology and did not provide any relief.

**Figure 4:**
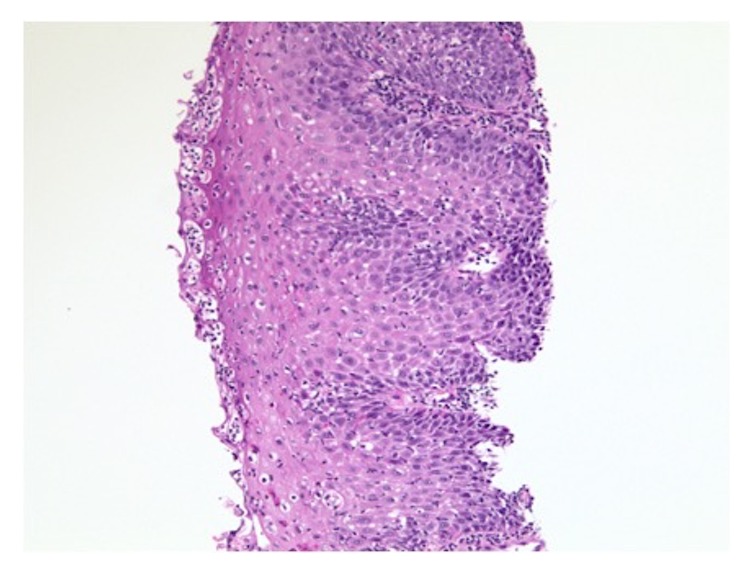
Esophageal squamous epithelium with H&E staining at 100x magnification. Findings showing lymphocytic infiltrate in the basal layer and scattered apoptotic keratinocytes (Civatte bodies).

**Figure 5:**
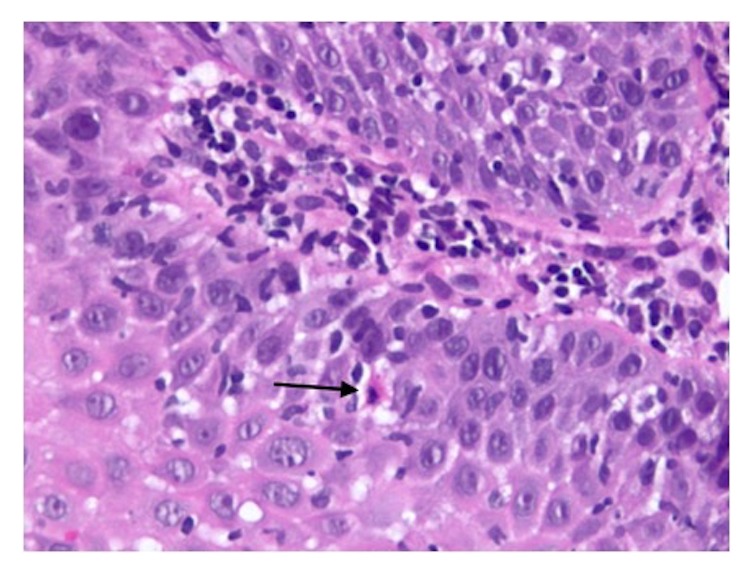
Esophageal squamous epithelium with H&E staining at 200x magnification with black arrow showing a Civatte body.

Seven years after her initial presentation, deep esophageal biopsy specimens were obtained showing severe acute and chronic esophagitis with a lichenoid-like pattern of chronic inflammation with notable features of lymphocytic infiltrate involving the basal layer of the epithelium and scattered apoptotic keratinocytes (Civatte bodies) (Figures 4 and 5) raising strong consideration of ELP. After a multidisciplinary review of these findings along with her consistent history, a diagnosis of ELP was made.

**Figure 6:**
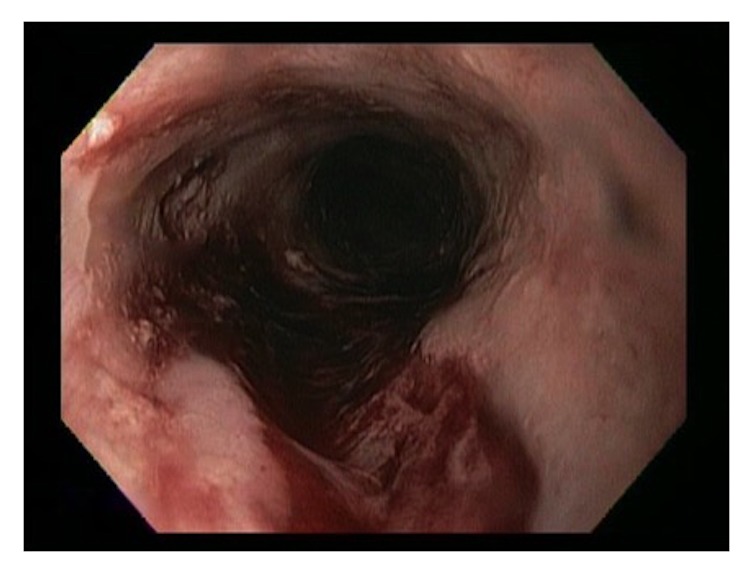
Esophageal mucosal tear after dilatation procedure occurring late in the patient's course highlighting the potential increased risk of complications with further endoscopic interventions.

By this time, her symptoms had already become severely debilitating with worsening strictures and narrowing throughout the esophagus, carrying a substantial risk for perforation with continued dilations (Figure 6). The patient ultimately made a decision to undergo a minimally invasive esophagectomy for definitive management with acute and chronic inflammation seen on surgical pathology. Her symptoms significantly improved in the months following her surgery. However, she gradually developed symptoms of dysphagia, abdominal cramping, nausea, emesis, and weight loss. This was felt to be secondary to delayed gastric emptying and a mild anastomotic stricture. She underwent Botox injection into the pylorus along with therapeutic dilation and triamcinolone acetonide injection to stricture. Her symptoms improved after these interventions.

## 3. Discussion

Similar to our case, the majority of patients with ELP are middle-aged to elderly females (approximately 87% females with a median age of 61.9) [2]. Dysphagia is the most common symptom and was the predominant complaint in this case [3, 5]. Patients often have a history of LP but ELP may be the initial manifestation. Although some autoimmune diseases have been reported to be associated with LP [1], the reported coexisting diseases in ELP are limited in several reports. There has been though a noted increased history of thyroid disorders in ELP cases [2]. Although hepatitis C has been associated with LP, it is infrequent in ELP reports, consistent with our findings [2, 3].

On endoscopic examination, ELP may involve the proximal to distal esophagus; however the proximal esophagus (estimated 89% of cases) is most often affected. This is in contrast to reflux disease with distal involvement [2]. Macroscopic findings are nonspecific but include a friable mucosa, whitish papules, esophageal webs, and strictures that can be multiple in numbers [3, 5, 6].

Histopathology may be nondiagnostic in half of cases with interpretations of esophagitis or chronic inflammation. Findings that help support a diagnosis of ELP include a band-like lymphocytic infiltrate involving the superficial lamina propria and basal epithelium along with the presence of Civatte bodies [7, 8]. Despite multiple biopsies in this case, a diagnosis of ELP was not suggested on histopathology until deep biopsies were performed.

As in our case, reflux esophagitis is often initially suspected with initiation of PPI therapy leading to no improvement in strictures [4]. ELP is often a diagnostic challenge with a large series [3] showing a mean of nearly five years until diagnosis. As a result, multiple endoscopies and dilations are often performed before reaching a diagnosis [3, 4].

Treatment generally starts with oral glucocorticoid therapy. The suggested approach has been doses in the range of 40–60 mg for several weeks (patients usually show response within first couple weeks) with a tapered course [2, 8]. Though the majority of patients often respond to this approach, some may develop symptomatic recurrence during tapering and require longer therapy. In addition, those who had complete response often have recurrence of disease later on highlighting the potential chronic and recurring nature of ELP [4, 5]. Failure to respond is likely to occur for those with strictures at diagnosis as in our case [2]. Fluticasone propionate and intralesional triamcinolone acetate have also been utilized for improving symptoms [9, 10]. Aside from glucocorticoids, limited reports have utilized tacrolimus (including aqueous preparations) and cyclosporine with reported good response [10, 11]. Another aspect of treatment has been dilations for strictures in order to achieve immediate improvement in symptoms [5, 12]. Some cases have demonstrated concern for inducing the Koebner phenomenon at sites of trauma with dilatation [4]. However, dilatations are often necessary in patients and are performed with concurrent therapy aimed at controlling the disease [3]. Moreover, some patients even with prolonged and multiple treatment strategies may not respond sufficiently resulting in frequent dilatation procedures [8]. Our patient had developed poor responses despite aggressive steroid therapies and dilations, ultimately leading to definitive surgical management.

Although it is not certain if LP itself is an independent risk for malignant conversions, there are cases of esophageal squamous cell cancer in those with ELP [3, 5, 13, 14]. Therefore surveillance should be considered. Given the rarity of reported ELP, variable responses to treatment, and reports of malignancy, management may be better served utilizing a multidisciplinary approach. In summary, clinicians should consider ELP in the differential for dysphagia, especially for middle- to elderly-aged females, with refractory stricturing disease and without typical GERD symptoms or failure to respond to antireflux management. Awareness of the clinical history along with improved recognition and reporting of characteristic histopathology of ELP may lead to an earlier diagnosis and optimize outcomes.
